# Will an innovative connected AideSmart! app-based multiplex, point-of-care screening strategy for HIV and related coinfections affect timely quality antenatal screening of rural Indian women? Results from a cross-sectional study in India

**DOI:** 10.1136/sextrans-2017-053491

**Published:** 2018-10-15

**Authors:** Nitika Pant Pai, Jana Daher, HR Prashanth, Achal Shetty, Rani Diana Sahni, Rajesh Kannangai, Priya Abraham, Rita Isaac

**Affiliations:** 1 Department of Medicine, McGill University, Montreal, Quebec, Canada; 2 Division of Clinical Epidemiology, Research Institute of the McGill University Health Centre, Montreal, Canada; 3 Rural Unit for Health and Social Affairs (RUHSA), Christian Medical College, Vellore, Tamil Nadu, India; 4 Department of Clinical Microbiology, Christian Medical College, Vellore, Tamil Nadu, India; 5 Department of Clinical Virology, Christian Medical College, Vellore, Tamil Nadu, India

**Keywords:** multiplex, point-of-care, innovative, HIV, STBBI, antenatal care, cross-sectional study

## Abstract

**Objectives:**

In rural pregnant Indian women, multiple missed antenatal screening opportunities due to inadequate public health facility-based screening result in undiagnosed HIV and sexually transmitted bloodborne infections (STBBIs) and conditions (anaemia). Untreated infections complicate pregnancy management, precipitate adverse outcomes and risk mother-to-child transmission. Additionally, a shortage of trained doctors, rural women’s preference for home delivery and health illiteracy affect health service delivery. To address these issues, we developed AideSmart!, an innovative, app-based, cloud-connected, rapid screening strategy that offers multiplex screening for STBBIs and anaemia at the point of care. It offers connectivity, integration, expedited communications and linkages to clinical care throughout pregnancy.

**Methods:**

In a cross-sectional study, we evaluated the AideSmart! strategy for feasibility, acceptability, preference and impact. We trained 15 healthcare professionals (HCPs) to offer the AideSmart! strategy to 510 pregnant women presenting for care to outreach rural service units of Christian Medical College, Vellore, India.

**Results:**

With the AideSmart! screening strategy, we recorded an acceptability of 100% (510/510), feasibility (completion rate) of 91.6% (466/510) and preference of 73%. We detected 239 infections/conditions (239/510, 46.8%) at the point-of-care, of which 168 (168/239; 70%) were lab confirmed, staged and treated rapidly. Of the 168 confirmed infections/conditions, 127 were anaemia, 11 Trichomonas and 30 hepatitis B virus (HBV) (25 resolved naturally, 5 active infections). Four infants (4/5; 80%) were prophylaxed for HBV and were declared disease-free at 9 months. Recruited participants were young; mean age was 24 years (range: 17–40) and 74% (376/510) were in their second trimester. Furthermore, 95% of the participants were retained throughout their pregnancy.

**Conclusion:**

The AideSmart! strategy was deemed feasible to operationalise by HCPs. It was accepted and preferred by participants, resulting in timely screening and treatment of HIV/STIs and anaemia, preventing mother-to-child transmission. The strategy could be reverse-innovated to any context to maximise its health impact.

## Introduction

### Nature and significance of the local problem

In rural Indian women, multiple missed opportunities impede timely screening of sexually transmitted bloodborne infections (STBBIs) and conditions (anaemia) associated with HIV. Untreated missed infections risk mother-to-infant transmission and adverse outcomes (ie, congenital infection, stillbirth, preterm delivery or low birth weight). The prevalence of anaemia, a related condition, is about 50% in South Asian women and it increases susceptibility to STBBIs.^1^ Furthermore, in pregnancy, physiological demands for iron and vitamins pose a risk of cardiac complications and postpartum haemorrhage.^2^

Although public health facilities offer vertical screening programmes for STIs and anaemia, pregnant women are often shunted between facilities, while screening tests are often unnecessarily repeated and not recorded. Moreover, as a result of poor documentation that often gets lost in the health system, treatment plans are not executed, precipitating losses to follow-up. Anecdotally, only 12% of rural public hospitals offer essential antenatal services for HIV, one of the top STIs.^3^ Screening for other STBBIs is sporadic and performed only when women present for early pregnancy care, which is rare. Furthermore, rural women are unaware of the importance of timely screening for STBBIs. In sum, missed screening opportunities and gaps across the care continuum, from access to retention, translate to poor quality of antenatal care in rural India. Taken together, they result in poor management of STBBIs and anaemia.

Delayed detection of STBBIs underestimates their true disease burden. Reported seroprevalence estimates differ greatly for the Southern Indian region: for HIV, prevalence ranges from 0.33% to 3% regionally^4 5^ vs 0.6% to 0.9% nationally;^6^ for syphilis, 11%–12.9% regionally to 1.2%–1.8% nationally;^7^ for hepatitis B virus (HBV), they are at an endemic high of 4%–5% to 0.9%–11.2% nationally;^8^ hepatitis C virus (HCV) is at 1.03%;^9^ ﻿ *Trichomonas vaginalis* (TV) at 4% and 33%–89% for anaemia.^10^﻿

### Available knowledge

#### Rationale

In India, frontline healthcare professionals (HCPs) who serve their own communities as peer health navigators play a major role in plugging the gaps in service delivery and in raising health awareness regarding maternal and child health.^11 12^ ﻿ If executed properly, this could result in timely access to screening programmes during antenatal care, ultimately leading to the effective reduction of maternal morbidity and mortality and the prevention of mother-to-infant transmission. Besides, as rapid point-of-care tests (POCTs) have been found to be accurate and effective for screening/triage of STBBIs,^13–15^ ﻿ strategies including POCTs represent the ideal method to offer a potential solution to plug gaps in the care continuum for STBBIs (refer to online appendix 1).

10.1136/sextrans-2017-053491.supp1 Supplementary data

The global availability of mobile phone technologies for health, mHealth, has enabled greater provision of maternal and child health services for STBBI/HIV through the use of short message services (SMS) and novel mobile applications.^16–18^ ﻿ This mHealth approach has demonstrated success in delivery of reproductive health information, tracking HIV/AIDS care and adherence to antiretroviral treatment.^19–21^ ﻿ mHealth offered by HCPs has increased knowledge with respect to antenatal and neonatal care and catalysed maternal behaviour improvements with positive impact on the offspring’s health and growth.^22 23^ When integrated within a reproductive sexual health programme, such a promising approach could increase access to quality antenatal care in rural areas and optimise service delivery to hard-to-reach populations.

**Figure 1:**
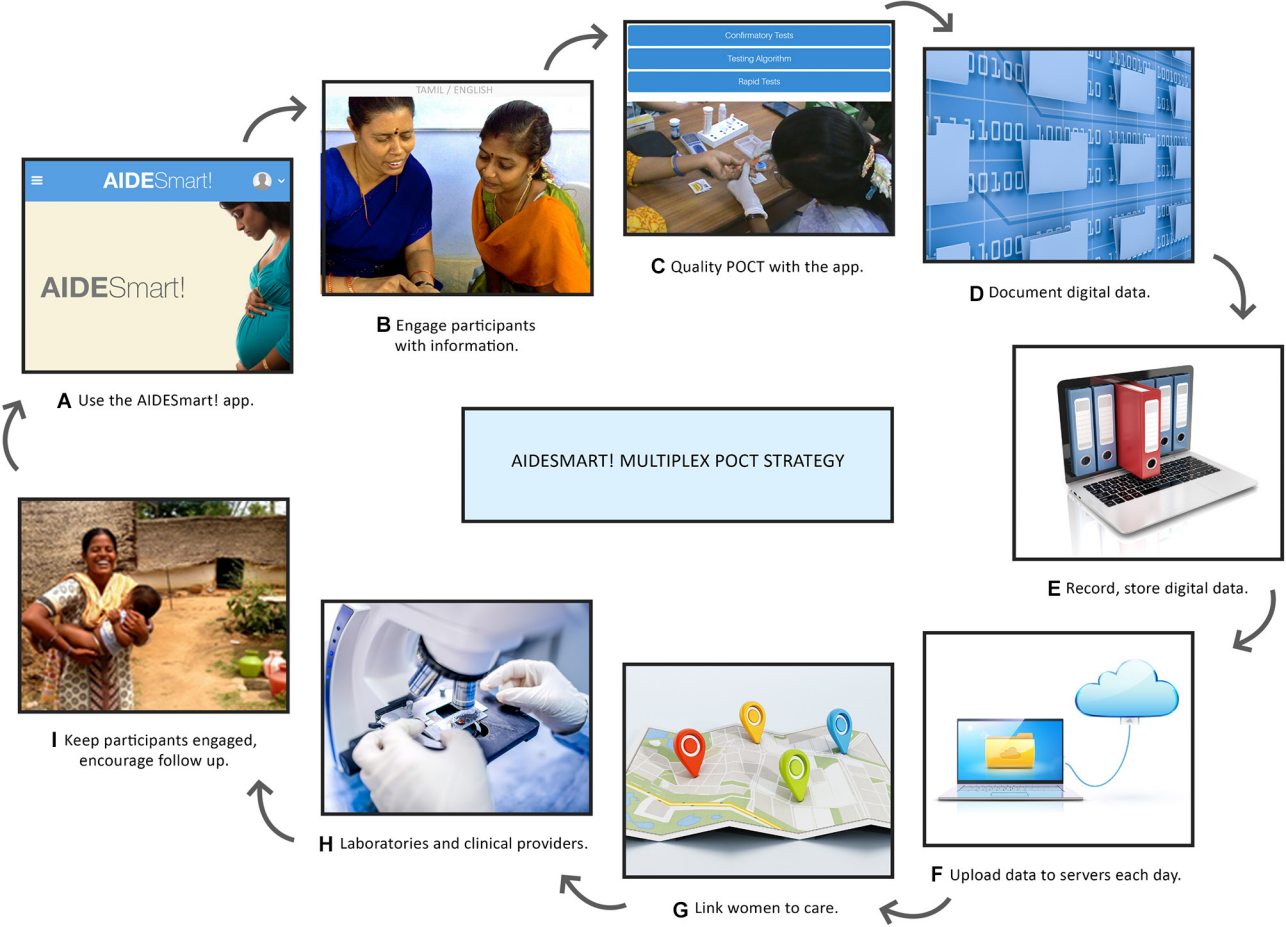
Complete AideSmart! multiplex POCT strategy performed by HCPs. HCPs, healthcare professionals; POCT, point-of-care test.

We hypothesised that our proposed integrated, digital, multiplex POCT-based AideSmart! (figure 1) strategy, operationalised by HCPs, that screens and links pregnant women to timely treatment and referral, could reduce the time to HIV/STBBI treatment initiation to possibly impact morbidity in women and transmission to their infants. It could additionally engage and empower patients and HCPs in the technology-driven delivery of connected care.

## Methods

### Context

A SmartApp-based, cloud-connected, multiplexed POCT strategy called AideSmart! (Nitika Pant Pai 2016, McGill University, Montreal Canada) was developed to plug gaps in engagement, timely screening and integrated clinical action and retention during STBBI screening and management (refer to online appendix 1).

#### Specific aims

In a cross-sectional study, we aimed to evaluate whether the AideSmart! strategy offered to participants by frontline HCPs could be feasible, acceptable and impact detection of new infections/conditions and retention in the care of women presenting for antenatal care in rural South India.

### Intervention

The AideSmart! strategy (figure 1) consisted of a secure, confidential, encrypted tablet application and linked web-based platform of care. We trained 15 HCPs to screen pregnant women with multiplexed POCTs and to facilitate care through the AideSmart! app. Each HCP followed 35 participants throughout their pregnancy and screened them close to their homes in rural service units. Services included: the provision of evidence-based information, multiplexed POCT quality control and assurance training, digital data collection and connectivity, rapid communication of results, lab integration and clinical follow-up. POCTs for the following infections/conditions were offered: HIV, HBV, HCV, syphilis, TV and anaemia. The HCPs used four POCTs: two rapid multiplexed finger-prick tests (Multiplo HBc/HIV/HCV test (anti-HBc total/anti-HIV-1/2/anti-HCV) and Multiplo TP/HIV test (anti-TP/anti-HIV-1/2)) and two rapid singleton tests (vaginal swab-based OSOM Trichomonas test (TV antigen) and finger-prick Hb test (Mission Hb) for anaemia (haemoglobin in g/L)).

HCPs were trained to perform the following tasks (figure 1): (1) use the AideSmart! app; (2) engage with participants, with evidence-based information (in English and Tamil); (3) conduct POCT, maintain quality control logs and extensively monitor the quality of POCT in harsh, field settings; (4) record digital data on each participant; (5) interpret, record, store and provide test results to lab professionals and providers; (6) upload data onto real-time servers at the end of each day; (7) track, communicate and link women to care with built-in links; (8) communicate with laboratories and engage with clinical providers and (9) convey clinical plans to participants, keep them engaged and updated, to encourage follow-up throughout their pregnancy.

### Study approach

**Figure 2:**
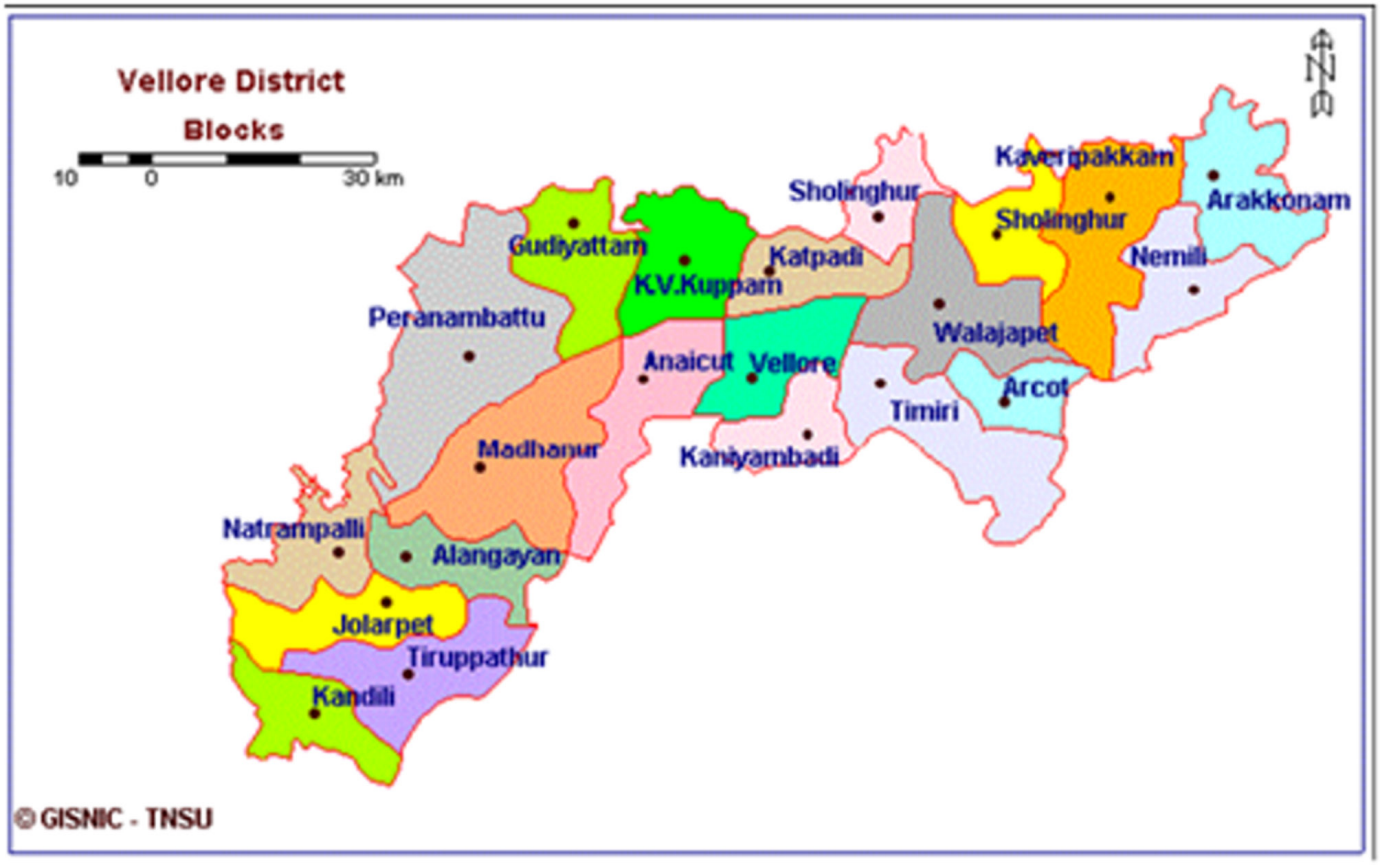
Map of Vellore district blocks.

The cross-sectional study was conducted during April 2015 to December 2016, in a convenient sample of 510 rural pregnant women, recruited from peripheral service units (PSUs). PSUs were spread around the K.V. Kuppam rural development blocks, in the Vellore district (figure 2). PSUs were managed by the Rural Unit for Health and Social Affairs (RUHSA), of the Christian Medical College (CMC) in Vellore, India.

#### Inclusion/exclusion criteria

Pregnant women aged 18 years old or above, able to provide informed consent, with access to a phone were eligible to participate. Women with a mental health or a serious medical condition requiring hospitalisation were excluded.

### Measures

#### Primary outcome

*﻿* *Feasibility* was defined by the metric completion rate, calculated as the proportion of participants successfully screened with POCTs and confirmatory tests (numerator), over the total number of consenting participants (denominator). With a hypothesised completion rate of 80%, with a CI width of 5%–10%, a sample size of 500 pregnant women was deemed sufficient for our estimations.

#### Secondary outcomes

*Acceptability* was defined as the proportion of pregnant women approached who consented to participate (numerator) over the number that presented for recruitment (denominator).

*Preference for the strategy* was defined as whether participants preferred POCTs over conventional laboratory tests. *Preference for referral* was defined as the willingness to recommend the strategy to a friend. *Preference for turnaround time* (TAT) was defined as the time to complete the tests and receive results. *Preference for the mode of follow-up* (ie, face-to-face, phone calls, SMS) and the average number of visits were documented.

*Participant satisfaction* was defined as the overall evaluation of the participant’s experience with the strategy, as measured by a Likert scale. Statements of HCPs and study participants were also recorded qualitatively.

#### Tertiary outcome

*Impact* was documented using proportions, defined as: (1) The number of new infections/conditions detected/confirmed relative to the total number of participants screened; (2) The number of linkages to confirmatory tests and treatment initiated over the number of patients that were detected with new infections/conditions; (3) The number of women who were retained in care/follow-up over the number of participants screened for all infections and (4) The number of infants in whom perinatal transmission was prevented, over the total number of infants delivered.

### The AideSmart! strategy

The HCPs were trained for 2–2.5 months on all study aspects. A pilot evaluation was conducted in 30 participants, with feedback provided to the HCPs in regard to their work flows and processes. Following this, the digital intervention was optimised and processes established to ensure a smooth running of the strategy (figure 1).

In order to ensure quality care throughout pregnancy, HCPs were trained to follow assigned pregnant women. Through the app, HCPs performed POCT, data and clinical tasks and engaged with each participant with information on the tablets. The importance of timely STBBI screening during pregnancy, and the need for engagement during treatment so as to prevent mother-to-infant transmission were explained. They communicated test results to the patients and maintained their engagement.

A laboratory team, consisting of a phlebotomist and a physician, provided an oversight of the POCT process as well as quality control and assurance. Repeat testing resolved discordant results, and all test results were documented and shared in real-time at the end of the day. With access to digital apps and servers, the results were available to all investigators involved and discussed regularly throughout the study period.

During the course of the study, approximately five prototypes of the AideSmart! app were developed and iterated; at multiple time points, bugs were removed and data server incompatibility issues were resolved. These modifications aided the creation of a refined version, resilient for use in a resource-constrained environment, with an improved connected app-based platform. For patients, video and patient-centred content was made available in their local language of Tamil, while for HCPs, data and study-based information was made available in both Tamil and English.

### Completeness and accuracy of data

With AideSmart!, the HCPs recorded deidentified and encrypted digital data on a secure platform. Data accuracy was verified on an ongoing basis by the project managers onsite and overseas. Data were communicated in real-time to all stakeholders (clinical providers, researchers, analysts, patients and laboratory professionals), using mobile technologies (SMS, phone calls, videos) to expedite management plans. The principal investigators arranged weekly teleconferences to troubleshoot plans, ensuring completeness and accuracy of the data. Data anonymity was ensured with high-level encryption and confidential password access. Servers were based and operated from Montreal.

### Analysis

#### Data sources and measurement

AideSmart! contained a semistructured validated questionnaire that collected deidentified and anonymised data on the following variables: sociodemographic, risk factor (table 1), medical gestational history, patient experience/satisfaction and diagnostic, clinical and follow-up data for each participant throughout their pregnancy, labour and delivery period.

**Table 1:** Demographic and risk factors data of participants

	N	%
Language		
Tamil	461	90.4
English	1	0.2
Telugu	43	8.4
Other	5	1.0
Education		
Illiterate	4	0.8
Primary school	8	1.6
Middle school	92	18.0
High school	282	55.3
Bachelor/Master or above	124	24.3
Number of children		
0 or 1	415	81.4
2 or 3	93	18.2
4 or 5	2	0.4
Status		
Unemployed	488	95.7
Worker	18	3.5
Student	4	0.8
Family monthly income		
<₹5000	182	35.7
₹5000–15 000	270	52.9
₹15 001–25 000	42	8.2
>₹25 000	16	3.1
Condom use		
Never	483	94.7
Always	1	0.2
Sometimes	26	5.1
Prior testing history		
HIV total	386	75.7
In the past 6 months	296	58.0
6 months to 1 year	9	1.8
>1 year	81	15.9
HBV total	157	30.8
In the past 6 months	109	21.4
6 months to 1 year	4	0.8
>1 year	44	8.6
HCV total	6	1.2
In the past 6 months	3	0.6
6 months to 1 year	0	0
>1 year	3	0.6
Syphilis total	283	55.5
In the past 6 months	214	42.0
6 months to 1 year	6	1.2
>1 year	63	12.4
Trichomonas	2	0.4
Anaemia	397	77.8

HBV, hepatitis B virus; HCV, hepatitis C virus.

### Quantitative data analyses

For all key quantitative outcomes (feasibility, acceptability, satisfaction, preference, impact), an analysis of proportions was performed.

### Clinical management

CMC’s protocols/algorithms for confirmatory testing were followed (online supplementary file 3). All infections/conditions were confirmed, staged and treated as per CMC protocols. The laboratories confirmed all POC test results. For Hepatitis B, molecular test-based referral and staging were performed. Regarding infants born to HBs Ag-positive mothers, ELISA HBsAg test was performed at 9 months and prophylaxis (hepatitis B immunoglobulin (HBIG) and vaccines) was administered. Metronidazole was administered for TV infection, while iron and folic acid were provided for anaemia management. All medications were documented on the application and medication reminders sent to the participant on a daily basis, keeping continuity of care.

10.1136/sextrans-2017-053491.supp3 Supplementary data

### Ethical considerations

The study investigators had neither perceived nor reported conflicts of interest with any intervention deployed in the study.

## Results

### Process measures and outcomes

Data on sociodemographics of participants revealed that participants were young; mean age: 24 years (range: 17–40; SD=4.0); educated: 80% (406/510) were high school graduates and poor: about 89% (452/510) with a monthly income of INR ₹15 000 (US$300). Seventy-four per cent of women (376/510) were in their second trimester.

#### Primary outcome

*Feasibility:* Feasibility of the AideSmart! strategy was documented at 91% (466/510).

#### Secondary outcomes

*Acceptability:* All 510 (100%) pregnant women approached for participation consented. *Preference:* Preference for POCT based screening strategy was high at 73% (359/492); preference for referrals was at 71% (349/492). Other preference measures were:

1. Preference for TAT for POCT test results: 1 hour—47% (232/492); 1 day—35% (174/492).
2. Preference for follow-up with in person visits: 47% (231/492).
3. Preference for follow-up on phone: 27% (131/492). The mean recorded frequency of in-person visits was 3.8 times (SD=1.6); 2.1 times by phone (SD=1.7) during pregnancy.

*Participant satisfaction:* 92% (453/491) of participants rated high satisfaction with the strategy.

#### Tertiary outcome

*Impact:* Baseline screening rates, observed in the past 6 months, by laboratory-confirmed estimates were low, with HIV at 58% (296/510); 42% for syphilis (214/510); 21% for HBV (109/510); 0.6% for HCV (3/510); 0.4% for TV (2/510) and 78% for anaemia (397/510) (table 1).

With AideSmart!, all consenting participants (100%) were screened at point-of-care; therefore, the incremental increase in screening from baseline was 42% for HIV, 58% for syphilis, 79% for HBV, 91.4% for HCV, 99.6% for TV and 28% for anaemia. At the point-of-care, 239 infections/conditions (46.8%; 239/510) were identified; 112 STIs and 127 cases of anaemia. Of 112 infections, the breakdown of new infections was as follows: 13 HIV, 69 anti-HBc, 11 syphilis, 18 TV and 1 HIV/HCV coinfection. Confirmatory testing of POCT results by CMC labs revealed that 168/239 infections/conditions (70%) were linked to care. Of those, 41 were infections and 127 anaemia. Lab confirmations revealed that all HIV POCT positives were negative (by Western Blot/ELISA) as well as the syphilis (confirmed by TPHA) and the HIV/HCV coinfection. Of 18 Trichomonas POCT positive results, 11 were shown to be positive as obtained by TV smear. All 127 anaemia cases were confirmed to be positive. Of 69 anti-HBc positive cases, 30 were confirmed; the infection resolved naturally in 34 patients. Five were HBs Ag-positive, of which four were referred to a hepatologist for treatment, while one was lost to follow-up, transmission was prevented in 4/5 infants (80%). All four women were further tested for HBV; DNA-reported viral load less than 2000 IU/mL, which was deemed ineligible for treatment as per CMC protocols. Their infants were prophylaxed with HBIG, vaccines at birth with three additional doses at 6, 10 and 14 weeks of age. They were each tested at 9 months with Elisa HBs Ag and found to be negative.

Medication intake was recorded diligently through AideSmart! by the HCPs. All (100%) TV-positive participants were administered Metronidazole. Nearly all (98%; 124/127) women with anaemia were treated with iron and folic acid supplements. At the end of the study, reported losses to follow-up were at 5% as 25 women chose to deliver in government facilities that incentivised labour and delivery services. With the strategy, 95.2% were retained in care (486/510). HBV seropositivity was at 1.07% (95% CI 0.44% to 2.55%); TV at 3.11% (95% CI 2.09% to 4.44%) and anaemia at 27.25% (95% CI 23.26% to 31.54%). These estimates are based on laboratory confirmation. Complete data were possible due to real-time documentation.

### Associations between intervention outcomes and contextual elements

Unintended consequences (refer to online appendix 1).

#### Missing data

Data were continuously updated in real-time and clinical issues were managed with teleconferences. Only 25 women (5%), who were paid (US$200) chose to deliver in government facilities and were lost to follow-up in our study.

## Discussion

### Key findings

With the AideSmart! strategy, screening rates for STBBIs and anaemia were greatly improved from baseline. All participants were rapidly screened/triaged for STBBIs with POCTs and 47% new infections were detected, of which 70% were rapidly linked to care. With a POCT-based strategy, increases in screening rates were highest for HBV, HCV, syphilis, TV, followed by HIV and anaemia. The participant retention rate was at 95%. As indicated by the metrics, the AideSmart! strategy generated good evidence with respect to feasibility, acceptance, preference, patient satisfaction and impact. The strategy improved the quality of POCT conduct, with a close monitoring of documentation and rapid confirmation of POCT results. Maintenance and recording of clinical data logs aided in quality control, monitoring and real-time surveillance of POCTs in challenging field conditions allowed instant troubleshooting.

AideSmart! was linked to cloud-based servers, through which POCT and lab-confirmed results were communicated to providers, researchers and patients in real-time, thusly allowing to effectively integrate POCT results with CMC labs and to deliver results to patients. A rapid TAT from POCT to confirmatory testing and treatment was maintained with integration of lab services at CMC and prompt actions were initiated by physicians, generating high participant and provider satisfaction. Through a close contact with the study participants, patient engagement, performed by HCPs, was high throughout the study, reducing the need for incentivisation. Furthermore, there were minimal losses to follow-up, at only 4.9%, (vs 15%–30% reported anecdotally). Finally, timely treatment and prophylaxis prevented HBV transmission in infants.

### Strengths

Our study design allowed for documentation of results cross-sectionally in real-time, which aided a complete documentation of data, processes and outcomes. Data quality and integration of results with laboratories were consistently maintained. POCT results were used to screen/triage patients and clinical management was initiated only after lab confirmed results were obtained. Our strategy is unique in that it offers a complete engagement, access, connectivity and retention solution, a step up from many promising digital innovative programmes that offer only one component of care, such as (1) CommCare, a digital data collection software program for frontline HCPs in Zambia;^24–26^ (2) eMOCHA (Johns Hopkins), a platform for real-time digital patient data;^27^ (3) SMS-based follow-up for HAART adherence/clinic attendance in Kenya;^28–30^ , ^w1^ (4) pilot connectivity for improved quality POCT testing, available in Zimbabwe.^w2-w7^

### Findings from our study

Regarding mother-to-child transmission, HBIG injections and immunisations (passive and active) were administered to infants of HBV-infected mothers who were born free of infection. Even with a high rate of unprotected sexual activity (95% unprotected intercourse), the absence of diagnosed syphilis, HCV and HIV infections may be due to a widespread use of antibiotics that eliminated syphilis and a lower exposure to unsafe injection practices (no HCV), that resulted in a decline in prevalence over time.

### Impact on people and systems

Enhanced communication aided by AideSmart! helped patients promote an active follow-up without financial incentives. Continuous monitoring kept stakeholders engaged at all times which impacted the quality of health service. Engagement has far-reaching consequences on subsequent care seeking and screening behaviour as qualitatively documented (box 1). For instance, one mother opined that she was unaware of the importance of screening for infections, but that she will actively ask for it in her next pregnancy.

Box 1 **Statements of healthcare professionals (HCPs) and study participants recorded in verbatim**

**HCPs**

*‘This project helped us to learn about the various infections in antenatal women as well as earnt to do the tests using the rapid test kits*.’

*‘As a health aide at the periphery, we were only entering data of antenatal mothers in the registers and following them up when they deliver. Through this project we learnt to do the rapid blood tests.’*

*‘I was confident to explain about these infections to the pregnant women.\ Moreover the additional salary support that I have got from the project was useful to my family.’*

*‘We got an opportunity to make good relationships with antenatal mothers by following them up more frequently every month.’*

*‘We learnt to educate pregnant women on what they should do during antenatal period. By telling them we also learnt it.’*

*‘This project was useful to me. Additional income that we have received was certainly an incentive, but more than that I felt happier to have learnt something that I can tell it to the next generations.’*

*‘I have heard about the infections only by its name but this project has taught me the effects of infections and how it can be prevented*.’

**Study participants**

*‘This project has helped me to detect the infections in me. I came to know how HepB is transmitted from mom to baby. I didn’t get tested for Hep B anywhere outside. If I was not tested here, it would have created a problem for my baby.’*

*‘This project was very useful because from our village all medical facilities are far but now I got tested here in my village. If not, we wouldn’t have got tested.’*

*‘It was useful and moreover it was free. So we got tested or else we can’t afford to get these tests done. Now my baby and I are fine.’*

*‘I’m hearing about these infections (Trichomonas) for the first time. When I heard that I have got the infection, at first, I was afraid but I got tablets from the RUHSA’s Sozhamur peripheral clinic and that helped me to have good health to deliver the baby. I want to do this test in the next pregnancy also.’*

*‘By this project, we came to know about the various infections and easy way of identifying it. Overall it was a useful one for me.’*

*‘Infections were identified by this test. If it was identified late, it would have been a shock and moreover the tests were done by RUHSA that made us feel safe and it did not cost us anything.’*

*‘At first when I got tested in an outside medical facility, they said that I didn’t have any infections but when we got tested through this project, they found that I have HepB infection. They also told me the ways to stop spreading it to the baby.’*

*‘I came to know that I have got the infection. I’m happy that we have got it for free.’*

*‘By this I came to know that I have got an infection and I got the tablets also. My husband doesn’t take me to any medical facility. After 3 years of marriage I have become pregnant. I think this time I will deliver the baby safely.’*

Observed and anticipated outcomes (refer to online appendix 1)

Opportunity costs (refer to online appendix 1)

### Limitations

Convenience sampling has a potential for selection bias and observational designs are limited by observed and unobserved confounding bias. We attempted to minimise verification, incorporation and detection biases by taking appropriate measures during the evaluation.

Usefulness of the work (refer to online appendix 1).

Sustainability (refer to online appendix 1).

#### Next steps

This AideSmart! strategy was recently funded by the Canadian Institutes of Health Research (CIHR), to be reverse-innovated to screen Canadian key populations such as men who have sex with men, injection drug users, immigrants and aboriginals across five provinces.

Additional references can be found in the online supplementary file.

10.1136/sextrans-2017-053491.supp2 Supplementary data

**Key messages**

- We evaluated the feasibility and impact of a digital SmartApp-based multiplexed integrated point-of-care test screening strategy for key STIs and anaemia in rural pregnant women.
- Fifteen healthcare workers were trained to offer the strategy close to participants’ homes. Acceptability and feasibility of the strategy were at 100% and 92%, respectively.
- The strategy impacted detection of 168 infections/conditions in pregnant women, prevented hepatitis B virus transmission to infants, with an overall loss to follow-up rate of 5%.
- In the next few years, the strategy will be reverse-innovated and scaled in Canadian provinces, while also transitioned to scale in Indian provinces.
