# Supplementary material for: Will an innovative connected AideSmart! app-based multiplex, point-of-care screening strategy for HIV and related coinfections affect timely quality antenatal screening of rural Indian women? Results from a cross-sectional study in India
Source: Sex Transm Infect. 2018 Oct 15;95(2):133–9. doi: 10.1136/sextrans-2017-053491 (PMC6580765; doi:10.1136/sextrans-2017-053491)
Supplement: Supplementary data [file sextrans-2017-053491supp001.pdf]

Available knowledge

In such settings, rapid point-of-care tests (POCTs) for STBBIs, with linked confirmatory testing and treatment, facilitated by digital innovations, could greatly improve the quality of antenatal screening. However, rapid POCT results suffer from incomplete documentation of POCT results, failed integration with labs, and poor communication that impedes timely clinical care. Besides, incomplete confirmatory test protocols with poor quality control further limits their diagnostic impact.[1-4] To improve service delivery, mobile health (mHealth) technologies could be leveraged by synergizing POCTs with mobile apps, expedited communication of POCT results with labs and with providers to initiate rapid clinical action.[5-12] In rural Asian and African settings, where physicians are hard to reach, peer-to-peer health navigation facilitated by skilled and semi-skilled frontline healthcare professionals (HCPs) is being deployed to improve health outcomes.[13] HCPs can be trained and empowered to offer digital POCT-based screening strategies.[14-16]

Context

Timely provision of high-quality antenatal care has downstream health implications, for both mothers and infants. Anecdotally, only 40% of HIV infected rural pregnant women report having received any antenatal care in India. This statistic reflects that facility-centred, laboratory-based, vertical screening programs for STBBIs have failed to meet screening and access needs of pregnant women. This problem is compounded by long commutes to healthcare centres, health illiteracy, preference for home delivery, and a disconnected system of health care.[17] Besides, a continued shortage of trained doctors in rural areas affects quality of rural health service delivery.[18] With treatment availability, there is a growing demand to expedite timely screening of STBBIs, so as to fast-track achievement of sustainable development goals (SDGs). In this

context, a patient friendly, lab-connected, innovative digital strategy that offered rapid and linked antenatal screening closer to women's homes offers a solution.

Associations between intervention outcomes and contextual elements

Analyses were restricted to documenting proportions on key outcome measures. No formal logistic regression analyses were performed thus no associations were therefore observed. Vellore is in a district with intermediate HBV endemicity, with an observed decline in HIV disease burden over time. Five pregnant mothers were proven to be HBV carriers; carrier rate of 1.07% was within the ranges (0.9-11.2%) reported previously.[19] The overall exposure to HBV (measured by anti-HBc) was low in this study, 6.4%, compared to 19.5% reported elsewhere in India.[20]

Unintended consequences

Table 2 illustrates the added value of the strategy as voiced by participants and providers. From the provision of evidence-based information with the agency of healthcare workers acting as peer navigators, the AideSmart! strategy helped increase participant engagement in their own care, and with digital communication, patients maintained a continued contact with the HCPs. This improved engagement throughout their pregnancy and study participants reported an increased awareness about infections, about screening with POCTs, and the need to care for themselves during pregnancy. Both the HCPs and clinical providers reported satisfaction with participant engagement, education and counselling.

Observed and anticipated outcomes

We did not anticipate such a high acceptability, feasibility, and patient satisfaction and impact in rural participants. Integration of lab results was possible with the excellent collaborations

between HCPs and CMC lab and clinical professionals that were further optimized with digital platforms. Strong data on feasibility suggests that the AideSmart! POCT strategy met the needs of patients, providers, research managers, and data analysts improving success rates. To the best of our knowledge, this is the first evaluation of a comprehensive SmartApp-based digital multiplexed STBBI POC screening strategy (that spans engagement to retention), and novelty contributed to its success. POCTs for Mission Hb initially underestimated the haemoglobin levels of some participants and misclassified them as anaemic. This discrepancy was attributed to the use of test strips validated in Chinese populations. The error was corrected, and test strips validated for Indian populations were subsequently used, improving our estimations.

Opportunity costs

We aim to evaluate the opportunity costs associated with the strategy in the near future, where we plan to scale the strategy across India.

Usefulness of the work

AideSmart! was successfully operationalized for feasibility, acceptability, preference by frontline HCPs in a resource challenged rural environment in India. It offers usefulness for similar resource constrained contexts and application through reverse innovations for settings that desire connected services.

Sustainability

Smart process innovations offer a potential to improve both the quality and efficiency of POCT processes. They plug gaps from screening, linkage, integration to impact health service delivery of STBBIs. They can be reverse innovated, adapted to many contexts and scaled. Integration with supply chain space can help generate a system wide impact on health care processes and

health care outcomes. Finally, smart public and private partnerships between POCT manufacturers, digital phone companies, and public health systems can help make them sustainable.

References

1. Engel N, Ganesh G, Patil M, et al. Point-of-care testing in India: missed opportunities to realize the true potential of point-of-care testing programs. *BMC Health Serv Res* 2015;15:550. doi: 10.1186/s12913-015-1223-3 [published Online First: 2015/12/15]
2. Bristow CC, Larson E, Javanbakht M, et al. A review of recent advances in rapid point-of-care tests for syphilis. *Sex Health* 2015 doi: 10.1071/SH14166 [published Online First: 2015/01/27]
3. Pai NP, Dhurat R, Potter M, et al. Will a quadruple multiplexed point-of-care screening strategy for HIV-related co-infections be feasible and impact detection of new co-infections in at-risk populations? Results from cross-sectional studies. *BMJ Open* 2014;4(12):e005040. doi: 10.1136/bmjopen-2014-005040 [published Online First: 2014/12/17]
4. Peeling RW, Mabey D. Point-of-care tests for diagnosing infections in the developing world. *Clin Microbiol Infect* 2010;16(8):1062-9. doi: 10.1111/j.1469-0691.2010.03279.x [published Online First: 2010/07/31]
5. Poorman E, Gazmararian J, Parker RM, et al. Use of text messaging for maternal and infant health: a systematic review of the literature. *Matern Child Health J* 2015;19(5):969-89. doi: 10.1007/s10995-014-1595-8 [published Online First: 2014/08/02]
6. Higgs ES, Goldberg AB, Labrique AB, et al. Understanding the role of mHealth and other media interventions for behavior change to enhance child survival and development in low- and middle-income countries: an evidence review. *J Health Commun* 2014;19 Suppl 1:164-89. doi: 10.1080/10810730.2014.929763 [published Online First: 2014/09/11]
7. Lee SH, Nurmatov UB, Nwaru BI, et al. Effectiveness of mHealth interventions for maternal, newborn and child health in low- and middle-income countries: Systematic review and meta-analysis. *J Glob Health* 2016;6(1):010401. doi: 10.7189/jogh.06.010401 [published Online First: 2015/12/10]
8. Akinfaderin-Agarau F, Chirtau M, Ekponimo S, et al. Opportunities and limitations for using new media and mobile phones to expand access to sexual and reproductive health information and services for adolescent girls and young women in six Nigerian states. *Afr J Reprod Health* 2012;16(2):219-30. [published Online First: 2012/08/25]
9. Lemay NV, Sullivan T, Jumbe B, et al. Reaching remote health workers in Malawi: baseline assessment of a pilot mHealth intervention. *J Health Commun* 2012;17 Suppl 1:105-17. doi: 10.1080/10810730.2011.649106 [published Online First: 2012/05/11]
10. L'Engle KL, Vahdat HL, Ndakidemi E, et al. Evaluating feasibility, reach and potential impact of a text message family planning information service in Tanzania. *Contraception*

2013;87(2):251-6. doi: 10.1016/j.contraception.2012.07.009 [published Online First: 2012/09/01]
11. Tamrat T, Kachnowski S. Special delivery: an analysis of mHealth in maternal and newborn health programs and their outcomes around the world. *Matern Child Health J* 2012;16(5):1092-101. doi: 10.1007/s10995-011-0836-3 [published Online First: 2011/06/21]
12. USAID, World Vision. Mobile technology stengthens behavior change communication and referrals by community health workers for maternal, newborn, and child health in rural Afghanistan 2014 [Available from: https://www.usaid.gov/sites/default/files/documents/1864/WorldVisionORBrief.pdf accessed 20 May 2016.
13. Karwa R, Maina M, Mercer T, et al. Leveraging peer-based support to facilitate HIV care in Kenya. *PLoS Med* 2017;14(7):e1002355. doi: 10.1371/journal.pmed.1002355
14. Meyers R, Kanfer R, Burrus C. Improving motivation and performance among frontline healthcare workers in rural India: the role of team-based goals and incentives. In: McWha-hermann I, Maynards D, O'Neill Berry M, eds. Humanitarian work psychology and the global development agenda; case studies and interventions. New York, NY: Routledge 2016:200-20.
15. Redick C, Faich Dini H. The Current State of CHW Training Programs in Sub-Saharan Africa and South Asia: What We know, What We Don't Know, and What We Need to Do. Washington DC: mPowering Frontline Health Workers, 2014.
16. Saprii L, Richards E, Kokho P, et al. Community health workers in rural India: analysing the opportunities and challenges Accredited Social Health Activists (ASHAs) face in realising their multiple roles. *Hum Resour Health* 2015;13:95. doi: 10.1186/s12960-015-0094-3 [published Online First: 2015/12/10]
17. Sinha G, Dyalchand A, Khale M, et al. Low utilization of HIV testing during pregnancy: What are the barriers to HIV testing for women in rural India? *J Acquir Immune Defic Syndr* 2008;47(2):248-52. [published Online First: 2008/03/15]
18. Bagchi S. Telemedicine in rural India. *PLoS Med* 2006;3(3):e82. doi: 10.1371/journal.pmed.0030082 [published Online First: 2006/03/03]
19. Geeta MG, Riyaz A. Prevention of mother to child transmission of hepatitis B infection. *Indian Pediatr* 2013;50(2):189-92. [published Online First: 2013/03/12]
20. Singh H, Aggarwal R, Singh RL, et al. Frequency of infection by hepatitis B virus and its surface mutants in a northern Indian population. *Indian J Gastroenterol* 2003;22(4):132-7. [published Online First: 2003/09/10]
