# Supplementary material for: Will an innovative connected AideSmart! app-based multiplex, point-of-care screening strategy for HIV and related coinfections affect timely quality antenatal screening of rural Indian women? Results from a cross-sectional study in India
Source: Sex Transm Infect. 2018 Oct 15;95(2):133–9. doi: 10.1136/sextrans-2017-053491 (PMC6580765; doi:10.1136/sextrans-2017-053491)
Supplement: Supplementary data [file sextrans-2017-053491supp003.pdf]

Web Tables

Web Table 1. Diagnostic accuracy of POC tests

	Sensitivity % [95%CI]	Specificity % [95%CI]	PPV % [95%CI]	NPV % [95%CI]
**Multiplo HBc/HIV/HCV**				
**HBV**	100 [88.4, 100]	92.9 [90.1, 95.1]	49.2 [36.1, 62.3]	100 [99.1, 100]
**HIV**	-	97.0 [95.0, 98.4]	-	-
**HCV**	-	99.8 [98.8, 100]	-	-
**Multiplo TP/HIV**				
**Syphilis**	-	97.9 [96.1, 99.0]	-	-
**HIV**	-	97.9 [96.1, 98.9]	-	-
**OSOM Trichomonas**	100 [71.5, 100]	98.5 [96.9, 99.4]	61.1 [35.7, 82.7]	100 [99.2, 100]
**Mission Hb anemia**	97.6 [93.3, 99.5]	40.4 [35.1, 45.8]	38.0 [32.7, 43.5]	97.9 [93.9, 99.6]

Web Table 2. Algorithm for confirmatory testing in pregnant women

	Positive POCT result	Negative POCT result
**HIV**	- 2 NACO rapid tests<br>- Western Blot for discordant rapid test results	- 1 NACO rapid test<br>- Western Blot for discordant rapid test results
**HBV**	- Enzyme immunoassay (EIA): testing for anti-HBc (similar to POCT) and HBsAg (recommended for pregnant women)<br>- DNA/RNA PCR for a positive EIA	- EIA: testing for anti-HBc and HBsAg<br>- DNA/RNA PCR for a positive EIA
**HCV**	- EIA: anti-HCV<br>- DNA/RNA PCR for a positive EIA	- EIA: anti-HCV<br>- DNA/RNA PCR for a positive EIA
**Syphilis**	VDRL+TPHA	RPR
**Trichomonas Vaginalis**	TV culture	Microscopy evaluation of vaginal wet mount
**Anemia**	Complete blood count	Complete blood count
