# Supplementary material for: Will an innovative connected AideSmart! app-based multiplex, point-of-care screening strategy for HIV and related coinfections affect timely quality antenatal screening of rural Indian women? Results from a cross-sectional study in India
Source: Sex Transm Infect. 2018 Oct 15;95(2):133–9. doi: 10.1136/sextrans-2017-053491 (PMC6580765; doi:10.1136/sextrans-2017-053491)
Supplement: Supplementary data [file sextrans-2017-053491supp002.pdf]

WEB REFERENCES

w1. Farmer T, Brook G, McSorley J, et al. Using short message service text reminders to reduce 'did not attend' rates in sexual health and HIV appointment clinics. *Int J STD AIDS* 2014;25(4):289-93. doi: 10.1177/0956462413502325 [published Online First: 2013/09/04]

w2. Chongo P, Sitoe N, Viegasa S, et al. Quality assurance for point-of-care testing in Mozambique's National Health Service. *African Journal of Laboratory Medicine* 2016;5(2):3 pages.

w3. Kebede A, Kebede Y, Desale A, et al. Quality assurance for point-of-care testing: Ethiopia's experience. *African Journal of Laboratory Medicine* 2016;5(4):5 pages.

w4. Cheng B, Cunningham B, Boeras D, et al. Data connectivity: A critical tool for external quality assessment. *African Journal of Laboratory Medicine* 2016;5(2):1-4. doi: 10.4102/ajlm.v5i2.535

w5. Alere. Alere connectivity for the Alere Prima CD4 2016 [updated 2016. Available from: http://www.alerehiv.com/ww/home/hiv-monitoring/alere-connectivity.html accessed December 16 2016.

w6. miDIAGNOSTICS. miDIAGNOSTICS selects emocha platform to embed miniaturized diagnostics into mobile health solution 2016 [updated 2016. Available from: http://www.midiagnostics.com/uploads/media/58244c65ed134/2016-11-07-midiagnostics-pr-collab-emocha-final.pdf?production-5ed4f01 accessed December 16 2016.

w7. QIAGEN. QIAGEN’s GeneReader NGS System streamlines data management for labs 2016 [updated 2016. Available from: http://www.genohm.com/genohm-co-develops-new-middleware-with-qiagen-for-genereader-ngs-system/ accessed December 16 2016.
